# Clinical significance of polyglutamylation in primary central nervous system lymphoma

**DOI:** 10.1186/s40478-018-0522-4

**Published:** 2018-02-23

**Authors:** Naoki Shinojima, Kenji Fujimoto, Keishi Makino, Kohei Todaka, Kazumichi Yamada, Yoshiki Mikami, Kazutaka Oda, Kazumi Nakamura, Hirofumi Jono, Jun-ichi Kuratsu, Hideo Nakamura, Shigetoshi Yano, Akitake Mukasa

**Affiliations:** 1 Department of Neurosurgery, Kumamoto University Hospital, 1-1-1 Honjo Chuo-ku, Kumamoto, 860-8556 Japan; 2 School of Medicine, Kumamoto University, Kumamoto, Japan; 3 Department of Diagnostic Pathology, Kumamoto University Hospital, Kumamoto, Japan; 4 Department of Pharmacy, Kumamoto University Hospital, Kumamoto, Japan; 5 Department of Neurosurgery, Sakurajuji Hospital, Kumamoto, Japan

**Keywords:** Polyglutamylation, Methotrexate, Primary central nervous system lymphoma, Leucovorin rescue

## Abstract

The therapeutic response to high-dose methotrexate (HD-MTX) therapy for primary central nervous system lymphoma (PCNSL) varies. Polyglutamylation is a reversible protein modification with a high occurrence rate in tumor cells. MTX incorporated into cells is polyglutamylated and strongly binds to dihydrofolate reductase without competitive inhibition by leucovorin (LV). Tumor cells with high polyglutamylation levels are selectively killed, whereas normal cells with lower polyglutamylation are rescued by LV. We hypothesized that the extent of polyglutamylation in tumor cells determines treatment resistance. Here, we investigated the therapeutic response of PCNSL to HD-MTX therapy with LV rescue based on polyglutamylation status. Among 113 consecutive PCNSL patients who underwent HD-MTX therapy in our department between 2001 and 2014, polyglutamylation was evaluated by immunostaining in 82 cases, with relationships between polyglutamylation and therapeutic response retrospectively examined. Human malignant lymphoma lines were used for in vitro experiments, and folpolyglutamate synthetase (FPGS), which induces polyglutamylation, was knocked down with short-hairpin RNA, and a stable cell line with a low rate of polyglutamylation was established. Cell viability after MTX treatment with LV rescue was evaluated using sodium butyrate (NaBu), a histone-deacetylase inhibitor that induces polyglutamylation by elevating FPGS expression. The complete response rate was significantly higher in the group with polyglutamylation than in the non-polyglutamylation group [58.1% (25/43) and 33.3% (13/39), respectively] (*p* < 0.05), and progression-free survival was also significantly increased in the group with polyglutamylation (*p* < 0.01). In vitro, the relief effect of LV after MTX administration was significantly enhanced after FPGS knockdown in al cell lines, whereas enhancement of FPGS expression by NaBu treatment significantly reduced this relief effect. These findings suggested that polyglutamylation could be a predictor of therapeutic response to HD-MTX therapy with LV rescue in PCNSL. Combination therapy with HD-MTX and polyglutamylation-inducing agents might represent a promising strategy for PCNSL treatment.

## Introduction

The standard treatment for primary central nervous system lymphoma (PCNSL) is high-dose methotrexate (HD-MTX)-based chemo-radiotherapies with leucovorin (LV) rescue [10, 16, 27]. The median overall survival of patients with PCNSL who undergo HD-MTX-based therapies is ~ 40 months [16, 27]; however, the therapeutic response to HD-MTX therapies varies in patients with PCNSL, with some cases showing poor therapeutic response or recurrence [21]. The established prognostic factors for therapeutic response to HD-MTX and for survival in PCNSL are age and performance status [2, 9], whereas no predictors have been identified for molecules supposedly targeted in this therapy. Polyglutamylation is a reversible post-translational modification of proteins that is thought to be involved in the stabilization of proteins, such as microtubules [7]. In contrast to normal cells, tumor cells show frequent occurrence of polyglutamylation [23]. Once the MTX transported into the tumor cells is polyglutamylated, it is retained and strongly binds to dihydrofolate reductase (DHFR) in a process that is not subject to competitive inhibition by LV, resulting in long-lasting inhibition of thymidylate synthase [14, 22, 24]. Therefore, MTX treatment can selectively kill cancer cells in which polyglutamylation has occurred, whereas normal cells with lower levels of polyglutamylation are rescued with LV [8]. In this context, we hypothesized that the therapeutic response to HD-MTX therapy with LV rescue is dependent upon the extent of polyglutamylation in PCNSL. This study investigated whether the extent of polyglutamylation could predict the response to HD-MTX therapy in patients with PCNSL. To the best of our knowledge, this is the first study revealing that polyglutamylation could be a significant predictor of the therapeutic response to HD-MTX therapy with LV rescue in PCNSL.

## Materials and methods

### For clinical investigation

#### Patients

**Fig. 1:**
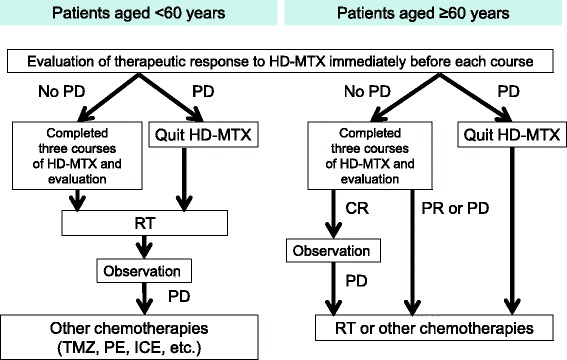
The PCNSL protocol used at our institute. A course of HD-MTX-based chemotherapy with LV rescue was administered every 3 weeks, with three such courses performed. Patients with PCNSL aged < 60 years were supposed to undergo RT from 4 weeks after the completion of three courses of HD-MTX treatment. Patients aged ≥60 years were monitored for CR to the therapy during follow-up evaluation after completion of the three courses. Alternatively, they underwent RT or other chemotherapies, such as temozolomide (TMZ), ICE (ifosphamide, cisplatin, and etoposide), or PE (carboplatin and etoposide). If PD occurred before the three courses of HD-MTX were completed, patients aged < 60 years underwent RT, whereas patients aged ≥60 years underwent RT or other chemotherapies. Second-line treatments were not uniform in cases of recurrence after HD-MTX therapies with or without RT. Patients who showed recurrence underwent TMZ, ICE, and PE treatment

After screening to rule out systemic lymphoma by positron emission tomography (PET) or whole-body computed tomography (CT), the patients with preoperative diagnosis of PCNSL exclusively located within the CNS underwent biopsy at our department. After confirming the histological diagnosis of PCNSL based on criteria published by the World Health Organization [20], HD-MTX therapy was conducted between January 2001 and December 2014 at our institute and affiliated hospitals according to our PCNSL protocol (Fig. 1). Paraffin-embedded tissues were obtained from 82 of the 113 patients who were newly evaluated with consecutive immunocompetent PCNSL. Under the approval for genetic and molecular analysis using patient specimens by the Research Ethics Committee of the Institutional Review Board of Kumamoto University Hospital [30, 32], this study was conducted after obtaining written informed consent from all participating patients or their family members.

#### PCNSL protocol

HD-MTX-based chemotherapy with LV rescue was performed according to our previously reported protocol [21]. Induction therapy consisted of a cycle of high-dose MTX (3.5 g/m^2^) delivered intravenously over 3 h on days 1, 22, and 43. LV rescue was initiated 24 h after the start of MTX infusion, and 15 mg of LV was intravenously administered nine times every 3 h, followed by five times every 6 h. The repeated intravenous administration of LV was continued until MTX clearance (< 0.1 μM). Procarbazine (60 mg/m^2^) was administered orally on days 1 through 7, 22 through 28, and 43 through 49. The initial betamethasone treatment dose was tapered from 16 mg to 2 mg every 4 days. The first-line therapy did not include any rituximab therapy. One course was administered every 3 weeks, and three such courses were performed (Fig. 1). Patients with PCNSL and aged < 60 years were supposed to undergo radiotherapy (RT) from 4 weeks after the completion of three courses of HD-MTX therapy. By contrast, patients aged ≥60 years were monitored for complete response (CR) to the therapy during follow-up evaluation after completion of the three courses. Alternatively, they underwent RT or other chemotherapies. If the progressive disease (PD) occurred before the three courses of HD-MTX were completed, patients aged < 60 years underwent RT, whereas those aged ≥60 years underwent RT or other chemotherapies. The second-line therapy was not uniform in cases of recurrence after completing HD-MTX therapies with or without RT.

#### Evaluation of therapeutic response to HD-MTX

The therapeutic response to HD-MTX was evaluated using CT or magnetic resonance imaging (MRI), with contrast-enhancement according to the response criteria published by the International PCNSL Collaborative Group [1]. Evaluation of the therapeutic response to HD-MTX was performed just before the initiation of each course of HD-MTX, 3 weeks after the completion of the three courses, and during the follow-up period. We evaluated all patients every 3 months for the first 2 years and every 6 months thereafter. CR was assumed in cases of both CR and unconfirmed CR (CRu). For patients unable to complete the three courses of HD-MTX due to adverse events associated with MTX, MRI data obtained after the final HD-MTX treatment was used for the evaluation.

#### Immunohistochemistry (IHC)

**Fig. 2:**
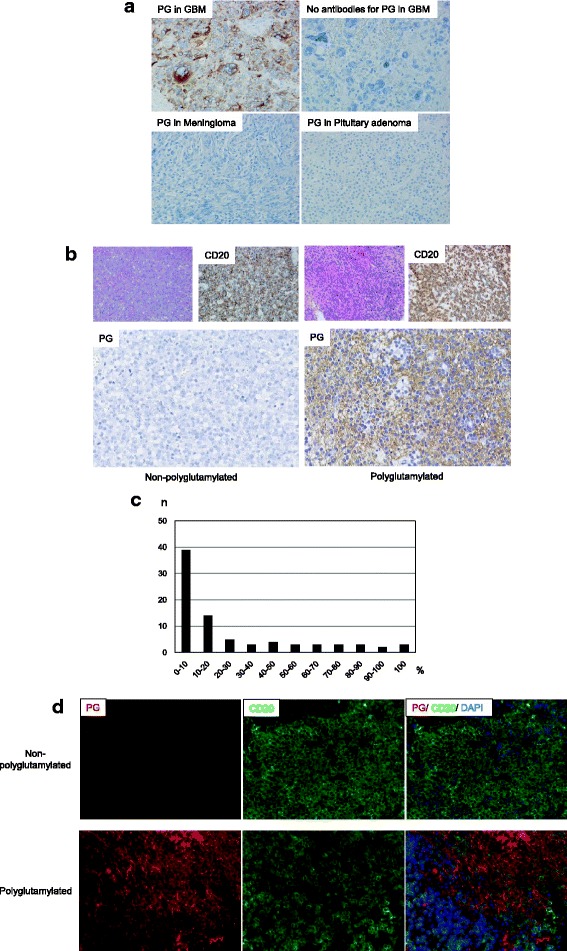
Representative figures of IHC for polyglutamylation. **a** GBM specimen used as positive control and stained for polyglutamylation (PG). Specimens of meningioma and pituitary adenoma, and the GBM specimen without primary antibodies were used as negative controls, revealing no staining for PG. Magnification, 200×. **b** Each subset of panels (left and right) shows polyglutamylated and non- polyglutamylated tumor specimens, respectively. The left upper panels are stained with hematoxylin and eosin, and the right upper panels show IHC results for CD20. Magnification, 200×. The bottom panels show IHC results for PG. Magnification, 400×. **c** Histogram showing the percentage of stained cells (10% cut-off). **d** Each subset of panels (upper and lower) shows polyglutamylated and non-polyglutamylated tumor specimens, respectively. Multiple-fluorescence staining using two different antibodies was performed. PG (red; left), CD20 (green; middle), and the merged fluorescence image (right), including DAPI (blue). After subtraction of background of autofluorescence, double-positive staining for both PG and CD20 revealed cytoplasm with a yellow-to-orange color as a result of overlapping fluorescence in the merged image in the polyglutamylated group. The red-fluorescent region (lower right) in the merged image of the polyglutamylated tumor specimens was considered brain parenchyma

IHC was performed on formalin-fixed paraffin-embedded (FFPE) tumor specimens with validation of positive and negative controls according to our previously reported protocol [29, 31]. The antibodies for polyglutamylation (mouse monoclonal; GT335; AdipoGen AG, Liestal, Switzerland) were used at 1:2000 dilution. A glioblastoma (GBM) specimen was used as a positive control for polyglutamylation, and specimens of meningioma and pituitary adenoma were used as negative controls, as was a GBM specimen without primary antibody treatment (Fig. 2a). Polyglutamylation positivity was quantified by manual counting. The antibodies for CD20 (rabbit monoclonal; SP32; Spring Bioscience, Pleasanton, CA, USA), a common B cell lymphoma marker, were used at 1:100 dilution. To classify the cell of origin of diffuse large B cell lymphoma into the germinal center B-cell-like (GCB) or non-GCB group using the algorithm established by Hans et al. [15], antibodies against the following proteins were used: CD10 (mouse monoclonal; 56C6; Leica Biosystems Newcastle Ltd., UK) at 1:25 dilution, Bcl-6 (mouse monoclonal; LN22; Leica Biosystems Newcastle Ltd., UK) at 1:100 dilution, and MUM1 (mouse monoclonal; MUM1p; Dako Cytomation, Glostrup Denmark) at 1:50 dilution.

#### Immunofluorescence of tissue sections

Multicolored immunofluorescence using different primary antibodies for polyglutamylation (AdipoGen) and CD20 (Spring Bioscience) was performed as reported previously [29]. To subtract the autofluorescence in FFPE tumor specimens, a Mantra system was used (PerkinElmer, Waltham, MA, USA) [25].

#### Area under the concentration-time curve of MTX (AUC_MTX_)

According to population pharmacokinetic analysis using the nonlinear mixed-effects modeling program (NONMEM, version 7.3.0) [17], we examined AUC_MTX_ in 67 patients with PCNSL whose plasma MTX concentrations were available. We also assessed the correlation between clinical response and AUC_MTX_ according to polyglutamylation status.

### Survival analysis

As previously reported [21], overall survival (OS) was measured as the time from initial diagnosis to death from any cause, and progression-free survival (PFS) as the time from diagnosis to the first PD. Cases in which extracranial lesions were found by PET or contrast-enhanced CT after treatment were also considered PD. Patients whose day of death was uncertain and patients who were alive on the day of analysis were censored, with the time from the first diagnosis to the last physical interaction or clinic visit used as the censoring time. Patients whose day of PD was uncertain and patients without PD on the day of analysis were censored with the time from diagnosis to that of the last MRI/CT showing a response. We investigated whether polyglutamylation could be a predictor of OS and PFS using Kaplan-Meier survival curves and multivariate analysis. For survival analyses, we used the log-rank test to compare the Kaplan-Meier curves for OS or PFS in patients who did and did not manifest polyglutamylation. To estimate the treatment response to MTX, we applied the Cox proportional-hazards model. Univariate analysis was used to estimate the prognostic relevance of polyglutamylation status, a predictive marker for HD-MTX treatment, and of patient age, sex, preoperative Karnofsky performance status (KPS), MSKCC prognostic scoring [2], cell of origin (GCB vs non-GCB) [15], lactate dehydrogenase (LDH) levels, and tumor location, in which the tumors were divided into deep (corpus callosum, basal ganglia, brain stem, and cerebellum) or non-deep location included in the International Extranodal Lymphoma Study Group score [9]. The variables were included in the Cox model and subjected to multivariate analysis.

### For experimental investigation

#### Cell lines

Human lymphoma cell lines, namely, HKBML, a human PCNSL-derived cell line; RAJI, a Burkkit lymphoma cell line; and TL-1, a lymph node B-lymphoma cell line, were purchased from RIKEN BioResource Center (Tsukuba, Japan). These floating cells were maintained as previously reported [26].

### Knockdown of folpolyglutamate synthetase (FPGS) in lymphoma cells

FPGS induces the accumulation of high levels of MTX polyglutamates in childhood leukemia [28]. To decrease this accumulation in lymphoma cell lines, we established stable, genetically modified cell lines in which FPGS was knocked down by a lentivirus system. Lentiviruses were prepared as reported previously [29]. PMD2.G (envelope vector) and psPAX2 (packaging vector) were purchased from Addgene (Cambridge, MA). FPGS-specific short-hairpin (sh)RNA constructs in pLKO.1 vectors were also obtained from Dharmacon (Lafayette, CO, USA), and an empty vector was used as a scrambled-sequence control. Cells were selected and maintained with puromycin (0.5–1 μg/mL).

### Cell viability assay

We used the Cell Counting Kit-8 (Dojindo Molecular Technologies, Inc., Kumamoto, Japan) to evaluate cell viability after individual treatment, as previously reported [3, 4]. Cells were treated with 100 nM MTX (Wako Pure Chemical Industries, Ltd., Osaka, Japan) for 24 h, followed by the addition of LV (Pfizer Japan, Inc., Tokyo, Japan) at a final concentration of 3 μg/mL and culturing for an additional 24 h. Cell viability assay was performed 48 h later. Histone-deacetylase inhibitors (HDACIs) enhance the antitumor effect of MTX by upregulating FPGS expression, thereby causing intracellular accumulation of long-chain MTX polyglutamates in childhood acute lymphoblastic leukemia (ALL) [19]. Sodium butyrate (NaBu; Sigma-Aldrich, St Louis, MO, USA), a pan-HDACI, was used in this study. Lymphoma cell lines were treated with 100 nM MTX with or without 1 mM NaBu for 24 h prior to the addition of LV. Cell viability was assessed 48 h later.

### Western blot

Western blot was performed as previously described [29]. The primary antibodies used were anti-FPGS (1:1000; rabbit polyclonal; Spring Bioscience), anti-DHFR (1:10,000; rabbit monoclonal; EPR5285; Abcam, Cambridge, MA, USA), and anti-α-tubulin (1:5000; mouse monoclonal; Sigma-Aldrich).

### Immunofluorescence of lymphoma cells

Lymphoma cells were collected, attached to glass slides using the cytospin method, and processed for immunofluorescence as previously reported [4, 29]. To evaluate polyglutamylation levels in cells, anti-polyglutamylation antibodies (AdipoGen) were used at 1:2000 dilution. 4′,6-Diamidino-2-phenylindole (DAPI; FluoroPure grade; Thermo Fisher Scientific, Waltham, MA, USA) was used for counterstaining.

### Statistical analyses

Statistical differences were assessed by Mann-Whitney U test, chi-squared test, log-rank test, and Student’s t test. Differences were determined to be statistically significant if *p* < 0.05. The data were represented as the mean ± standard deviation (SD) of at least three replicates for each experiment. The Statistical Package for the Social Sciences (SPSS version 19; IBM corp., Armonk, NY, USA) was used for all statistical analyses.

## Results

### Clinical investigation

Among 113 consecutive patients with PCNSL, sufficient tissue specimens were available from only 82 patients. There were no differences in the clinical characteristics of these 82 patients or the remaining 31 patients (data not shown). The 82 patients comprised 46 males and 36 females, with a median age of 67 years. The median KPS was 40 (range, 20–100). The rate of CR to HD-MTX was 46.4%, and median OS was 1275 days (~ 42.5 months). Five patients who responded to HD-MTX therapy switched to RT before completing three courses of HD-MTX because HD-MTX caused adverse events. Six patients who showed new extracranial lesions after treatments were considered PD, although they showed no intracranial lesions. Two patients died due to adverse events associated with HD-MTX, such as hemo-phagocytic syndrome and interstitial pneumonia. One of them was a responder, as evidenced by MRI results, and was censored regarding PFS.

Representative figures of IHC for polyglutamylation are shown in Fig. 2b. The histogram categorizing the percentage of cells stained for polyglutamylation into every 10% is shown in Fig. 2c. In the specimens of 30 patients, 0% of the cells were stained, whereas 0.5 to 2.5% of the cells were stained in the specimens of 9 patients. In the specimens of the other 43 patients, ≥10% (the average was 45%, range was 10–100%) of the cells were stained. Next, we examined the distribution of patients with CR and non CR at different cut-off values for positivity of polyglutamylation (Table 1). There was a correlation between therapeutic response to HD-MTX and polyglutamylation positivity at 10% cut-off. We defined the polyglutamylation group as having ≥10% positivity and the non-polyglutamylation group as having < 10% positivity in this study. Because the brain parenchyma could contribute to the observed polyglutamylation staining, we utilized a multiple-fluorescence system using two different antibodies for polyglutamylation and CD20 in a subset of samples from 6 patients (one was non-polyglutamylation, 5 were polyglutamylation group). After subtraction of the autofluorescent background, double-positive stained cells for both polyglutamylation and CD20 (exhibiting cytoplasm with a yellow-to-orange color as a result of overlapping fluorescence in the merged fluorescent image) were observed in the polyglutamylated group (Fig. 2d). These results confirmed that the CD20-positive lymphoma cells had a high level of polyglutamylation in the samples from the polyglutamylation group (Fig. 2d).

**Table 1:** Distribution of patients with CR and non CR at different cut-off values of polyglutamylation percentage

	CR	No CR	*p*
1% of cut-off value			
Polyglutamylation, ≧ 1% (*n* = 48)	26	22	
Non polyglutamylation, < 1% (*n* = 34)	12	22	0.091^a^
10% of cut-off value			
Polyglutamylation, ≧ 10% (*n* = 43)	25	18	
Non polyglutamylation, < 10% (*n* = 39)	13	26	0.025^a^
20% of cut-off value			
Polyglutamylation, ≧ 20% (*n* = 28)	16	12	
Non polyglutamylation, < 20% (*n* = 54)	22	32	0.16^a^
30% of cut-off value			
Polyglutamylation, ≧ 30% (*n* = 24)	13	11	
Non polyglutamylation, < 30% (*n* = 58)	25	33	0.36^a^

*CR* complete response

^a^Chi-squared test

The number of patients with CR, CRu, PR, and PD was seven, 18, five, and 13, respectively, in the polyglutamylation group and five, eight, seven, and 19, respectively, in the non-polyglutamylation group. The rate of GCB was 41.5% in the polyglutamylation group, which was significantly higher (*p* < 0.05) than that (17.9%) in the non-polyglutamylation group (Table 2).

**Table 2:** Clinical characteristics of patients based on polyglutamylation status

	Polyglutamylation (*n* = 43)	Non polyglutamylation (*n* = 39)	*p*
Age (y)			
Median (range)	68 (37–84)	67 (46–86)	0.70^*^
Sex (# of pts)			
Male/ Female	27/ 16	19/ 20	0.20^**^
KPS (%)			
Median (range)	50 (20–100)	40 (20–100)	0.43^*^
(# of pts) High (≥70) / Low (≤60)	12/ 31	10/ 29	0.82^**^
MSKCC prognostic score			
Median (range)	3 (1–3)	3 (1–3)	0.76^*^
Cell of origin			
GCB/ non-GCB	17/ 24^a^	7/ 32	0.022^**^
LDH (# of pts)			
High/ Normal range	16/ 27	15/ 24	0.91^**^
Location (# of pts)			
Deep/ No	35/ 8	27/ 12	0.20^**^

*KPS* Karnofsky performance status, *pts.* patients

^*^ Mann-Whitney *U* test

^**^ Chi-squared test

^a^ data from two patients were not available

**Fig. 3:**
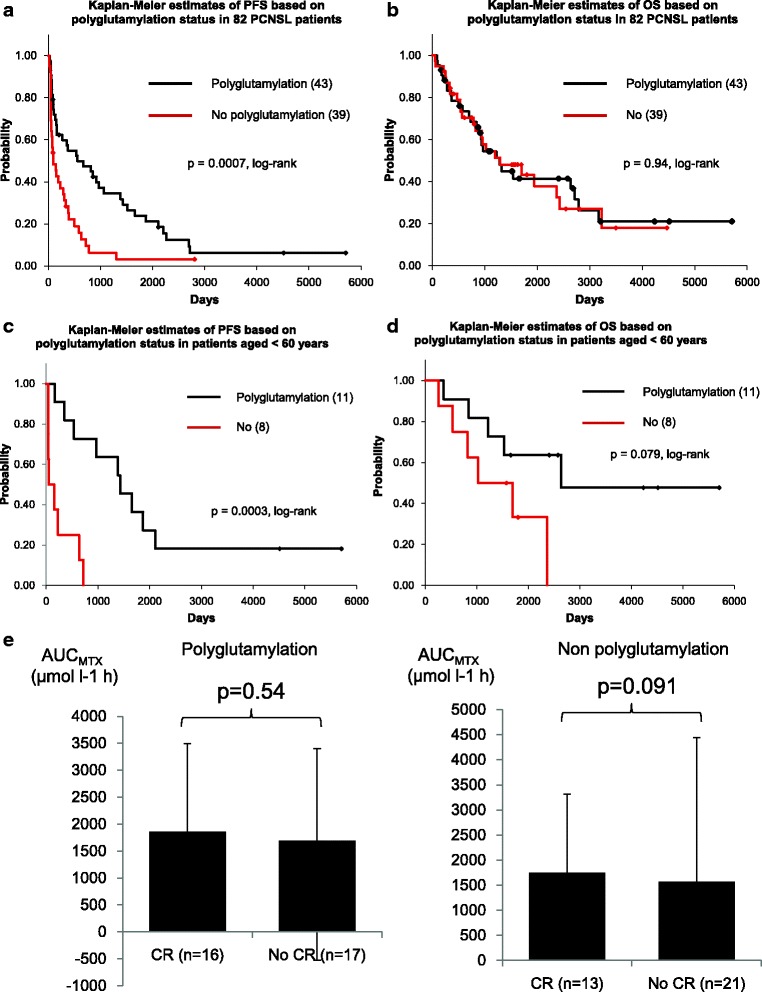
Kaplan-Meier curves of (**a**) PFS and (**b**) OS based on polyglutamylation status in 82 PCNSL patients. **c** PFS and (**d**) OS based on polyglutamylation status in 19 PCNSL patients aged < 60 years. **e** Correlation between clinical response and AUC_MTX_ according to polyglutamylation status

The median PFS was 560 days in the polyglutamylation group and 95 days in the non-polyglutamylation group. Kaplan-Meier curves confirmed that PFS was significantly longer in the polyglutamylation group than in the non-polyglutamylation group (*p* < 0.01; Fig. 3a), whereas there was no difference in OS between the two groups (Fig. 3b). Multivariate analyses confirmed that polyglutamylation status was the only significant independent predictor for PFS (*p* < 0.01; Table 3), although it was not a prognostic factor for OS (Table 4). We then determined the prognostic value in patients with PCNSL aged < 60 years, because they had received similar treatments, i.e., RT following HD-MTX, as part of their first-line therapy with or without PD other than older patients. Similarly, PFS was significantly longer in the polyglutamylation group relative to the non-polyglutamylation group in patients aged < 60 years (*p* < 0.01; Fig. 3c). Moreover, we found a tendency toward a better median OS in the polyglutamylation group as compared with that in the non-polyglutamylation group for patients aged < 60 years (*p* = 0.079; Fig. 3d). These results suggest that the polyglutamylation status in PCNSL tissues could be a predictor of therapeutic response to HD-MTX.

**Table 3:** Cox proportional hazard model for PFS

	Univariate analysis		Multivariate analysis	
Variable	HR (95% CI)	*p*	HR (95% CI)	*p*
Age	1.015 (0.995–1.036)	0.14	1.005 (0.972–1.038)	0.79
KPS (high vs. low)	1.328 (0.784–2.250)	0.29	0.717 (0.340–1.513)	0.38
Sex (M vs. F)	1.531 (0.953–2.457)	0.078	1.445 (0.820–2.545)	0.20
MSKCC score	1.151 (0.819–1.619)	0.42	0.782 (0.410–1.491)	0.46
Cell of origin (GCB vs. non-GCB)	0.696 (0.407–1.191)	0.19	1.086 (0.576–2.048)	0.80
LDH (high vs. normal range)	1.306 (0.801–2.129)	0.29	1.532 (0.860–2.731)	0.15
Location (deep vs. not deep)	0.928 (0.531–1.622)	0.79	1.377 (0.732–2.587)	0.32
Polyglutamylation vs. non-polyglutamylation	0.439 (0.269–0.716)	0.0010	0.461 (0.259–0.823)	0.0087

*PFS* progression-free survival, *HR* hazard ratio, *CI* confidence interval, *KPS* Karnofsky performance status, *M* male, *F* female

**Table 4:** Cox proportional hazard model for OS

	Univariate analysis		Multivariate analysis	
Variable	HR (95% CI)	*p*	HR (95% CI)	*p*
Age	1.027 (1.002–1.053)	0.035	1.047 (1.005–1.092)	0.028
KPS (high vs. low)	0.376 (0.182–0.779)	0.0085	0.263 (0.096–0.724)	0.0097
Sex (M vs. F)	1.096 (0.617–1.945)	0.75	0.712 (0.369–1.374)	0.31
MSKCC score	1.534 (0.982–2.394)	0.060	0.607 (0.277–1.334)	0.21
Cell of origin (GCB vs. non-GCB)	0.914 (0.483–1.730)	0.78	1.129 (0.546–2.334)	0.74
LDH (high vs. normal range)	1.029 (0.563–1.881)	0.93	0.773 (0.391–1.528)	0.46
Location (deep vs. not deep)	0.796 (0.419–1.514)	0.49	0.603 (0.297–1.226)	0.16
Polyglutamylation vs. non-polyglutamylation	0.978 (0.557–1.718)	0.94	1.728 (0.716–4.171)	0.22

*OS* overall survival, *HR* hazard ratio, *CI* confidence interval, *KPS* Karnofsky performance status, *M* male, *F* female

### Correlation of clinical response between polyglutamylation status and AUC_MTX_

In the non-polyglutamylation group, 33.3% (13/39) of the patients had CR (Table 1). The MTX concentrations might be an underlying factor associated with the observed differences in clinical response, as AUC_MTX_ is an important outcome predictor [18]. We examined the correlation between clinical response and the AUC_MTX_ in 67 patients whose plasma MTX concentrations were available. The average AUC_MTX_ was 1705.7 (range, 1074.2–5754.2) μmol/L/h in the 67 patients, and there was a tendency toward the average AUC_MTX_ being higher in patients having CR as compared with those with no CR (1748.8 μmol/L/h vs.1568.3 μmol/L/h, respectively; *p* = 0.091; Fig. 3e) in the non-polyglutamylation group. However, in the polyglutamylation group, there was no correlation in average AUC_MTX_ between patients having CR and those having no CR (1863.4 μmol/L/h vs 1694.1 μmol/L/h, respectively; *p* = 0.54; Fig. 3e). This result might explain why one third of the patients showed CR in the non-polyglutamylation group.

### Experimental investigation

**Fig. 4:**
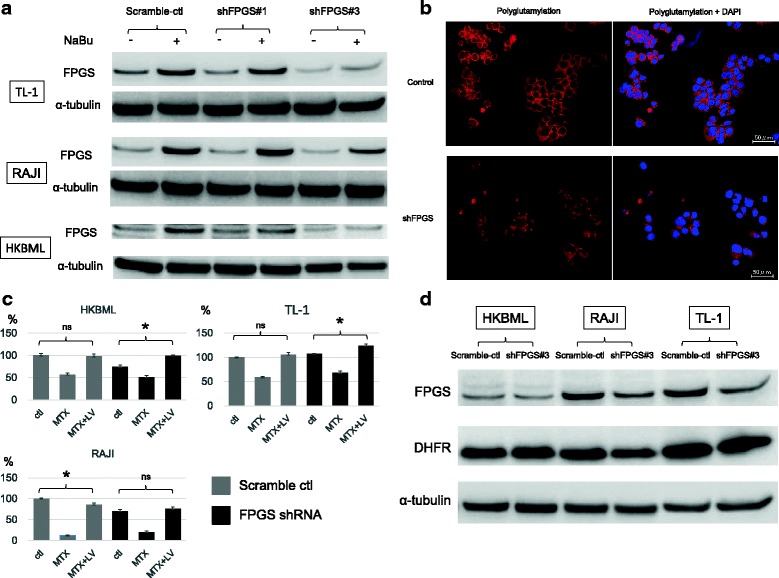
**a** Immunoblotting for FPGS in different cell lines (scramble control and shFPGS#3) with or without exposure to 1 mM NaBu for 72 h. α-Tubulin was used as the internal control. **b** Immunofluorescence to identify polyglutamylation in TL-1 control and TL-1-shFPGS cells. Scale bar, 50 μm. **c** Cells were treated with MTX for 24 h, followed by the addition of LV and culturing for another 24 h. Cell viability assay was performed 48 h later. Error bars represent SDs. Viability of cells treated with MTX and MTX + LV as compared with that of control cells (no treatment). *p < 0.01. ns, not statistically significant. **d** Immunoblotting for DHFR in different cell lines (scramble control and shFPGS#3). α-Tubulin was used as the internal control

To confirm the clinical results, we performed an in vitro study. To prevent the accumulation of polyglutamylation in lymphoma cells, we established a stable cell line in which FPGS was knocked down using shRNA constructs. Western blot results showed that FPGS expression was decreased in all cell lines treated with shRNA construct #3 (shFPGS#3), even after NaBu treatment, as compared with scrambled-sequence control cells (Fig. 4a). We used cell lines in which FPGS was knocked down by shFPGS#3 for all subsequent analyses and used immunofluorescence to confirm decreased polyglutamylation in FPGS-knockdown cells. The immunofluorescence level of polyglutamylation in the cytoplasm of FPGS-knockdown cells was lower than that in scrambled-sequence control cells (Fig. 4b). We then examined cell viability after MTX treatment and LV rescue. In HKBML and TL-1 cells, MTX-treated scrambled-sequence control cells were rescued by LV to the same level as control cells without treatment. However, the relief effect of LV after MTX treatment was significantly enhanced in cells exhibiting lower polyglutamylation levels by FPGS knockdown as compared with that in scrambled-sequence control cells (Fig. 4c). In RAJI cells after MTX and LV treatment, the viability of scramble control cells was significantly lower than that of controls without treatment. Additionally, the viability of cells in which FPGS was knocked down was restored to levels similar to that of controls without treatment. These results suggested that lymphoma cells with low levels of polyglutamylation were resistant to HD-MTX therapy with LV rescue. Although the tight binding of polyglutamylated MTX to DHFR is not subject to competitive inhibition by LV, resulting in long-lasting inhibition of DHFR [14, 22, 24], it is possible that DHFR expression is related to polyglutamylation levels in cells. We evaluated the expression of DHFR in control cells and in FPGS-knockdown cells, finding no difference in DHFR expression (Fig. 4d). Therefore, these findings suggest that DHFR activity might be regulated by polyglutamylated MTX.

**Fig. 5:**
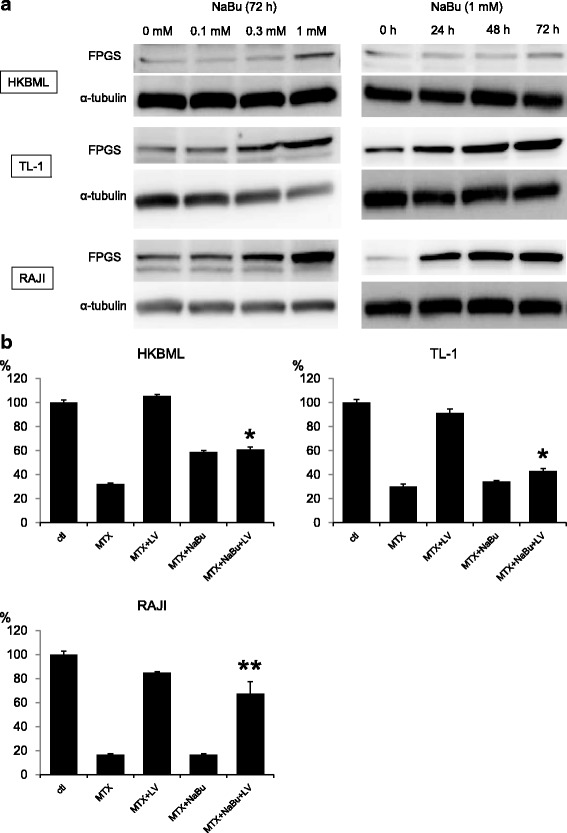
**a** Immunoblots showing upregulation of FPGS by NaBu treatment in a concentration- and time-dependent manner. α-Tubulin was used as the internal control. **b** Cells were treated with MTX with or without NaBu for 24 h, followed by addition of LV and culturing for another 24 h. Cell viability assay was performed 48 h later. Error bars represent the SD. Viability of cells treated with MTX + NaBu+LV as compared with that of control cells without NaBu treatment (MTX + LV). *p < 0.001; **p < 0.05

HDACIs upregulate FPGS, causing intracellular accumulation of long-chain MTX polyglutamates and increasing the efficiency of MTX therapy with LV rescue in childhood ALL [19]. Here, we examined whether HDACIs could enhance the antitumor effect of MTX with LV rescue in lymphoma cells using NaBu. FPGS expression was upregulated by NaBu treatment in a concentration- and time-dependent manner (Fig. 5a), and the relief effect of LV after MTX treatment was significantly reduced after NaBu treatment, resulting in an increased antitumor effect (Fig. 5b). Therefore, combination therapy involving HDACIs and HD-MTX with LV rescue might be more effective for patients with PCNSL via the FPGS/polyglutamate axis.

## Discussion

HD-MTX-based therapy with LV rescue is established as the standard treatment method for patients with PCNSL [10, 16, 27]. The therapeutic response to HD-MTX therapy varies, and no predictors for therapeutic response or survival have been identified in patients with PCNSL. MTX treatment with LV rescue can selectively kill tumor cells harboring high levels of polyglutamylation, whereas healthy cells with lower levels of polyglutamylation are rescued by LV [8]. We hypothesized that the therapeutic response to HD-MTX therapy with LV rescue depends upon the extent of polyglutamylation in PCNSL. We found that polyglutamylation is indeed a significant predictor of the response to HD-MTX therapy in patients with PCNSL. Additionally, our in vitro studies confirmed that the therapeutic response to HD-MTX treatment with LV rescue was dependent upon the extent of polyglutamylation in lymphoma cell lines, which was consistent with our clinical results. To the best of our knowledge, there have been no studies evaluating the correlation between therapeutic response and polyglutamylation in PCNSL. In other types of malignant neoplasms, high levels of MTX polyglutamylation (for example, in pediatric ALL, such as pediatric B cell lineage ALL) are correlated with therapeutic response, with pediatric B cell lineage ALL showing higher cure rates as compared with adult ALL and T cell lineage ALL [11]. The mechanism of resistance to MTX in leukemia possibly involves decreased uptake of the drug or lack of drug retention due mainly to low levels of polyglutamylation, increased polyglutamate breakdown, or increased DHFR activity [5, 6, 14]. Decreased expression of the reduced folate carrier, a transport protein, was associated with impaired MTX transport and was observed in relapsed acute lymphocytic leukemia after treatment with MTX therapy [13]. Low levels of DHFR gene amplification might also be an important cause of MTX resistance in ALL [12]. Such mechanisms of resistance to MTX aside from impaired polyglutamylation might explain why CR to MTX therapy was observed in only 60% of the patients with PCNSL who showed polyglutamylation in the present study. In contrast, one third of patients in the non-polyglutamylation group had CR. AUC_MTX_ might be a predictor for therapeutic response [18], and here, we found higher AUC_MTX_ values in patients with CR as compared with those with no CR. Therefore, the combination of polyglutamylation status and AUC_MTX_ might be useful for prediction of MTX therapeutic response.

Regarding prognostic factors for survival in patients with PCNSL, polyglutamylation status was the only statistically significant prognostic factor for PFS, but did not qualify as a significant predictor of OS. Age and performance status, which have already been established as predictors of OS, were the only significant prognostic factors for OS in this study. These results might be explained by the fact that both the first- and second-line treatments were heterogeneous among differently aged groups in this study. Therefore, we examined the correlation between polyglutamylation status and clinical outcome in patients aged < 60 years, all of whom received radiation as part of their first-line therapy. We found a tendency toward better median OS in the polyglutamylation group than in the non-polyglutamylation group (Fig. 3c). To investigate whether polyglutamylation status predicts OS, further analysis of PCNSL patients who have previously undergone homogeneous therapies is needed.

The multiple-fluorescence system revealed that in addition to tumor cells expressing CD20 and exhibiting high levels of polyglutamylation, brain parenchyma also exhibited polyglutamylation, as revealed by red color resulting from non-overlapping fluorescence in the merged image (Fig. 2d). Although we used a 10% cut-off value based on IHC positivity, we might be able to identify a more accurate cut-off value for clinical use through the multiple-IHC-staining platform.

HDACIs improve the efficiency of MTX therapy with LV rescue in childhood ALL by upregulating FPGS expression and causing intracellular accumulation of long-chain MTX polyglutamates [19]. Our results revealed that HDACIs reduced the relief effect of LV after MTX therapy, thereby enhancing the antitumor effect. Therefore, modification of polyglutamylation levels could improve the antitumor effect of HD-MTX-based therapies for PCNSL. Identifying new drugs or subjecting known drugs to drug repositioning to specifically upregulate polyglutamylation in tumor cells could improve the efficacy of PCNSL treatment.

### Limitations

This was a retrospective study, therefore, prospective studies on the extent to which polyglutamylation levels predict the therapeutic response to HD-MTX treatment with LV rescue should be performed.

## Conclusions

Our findings suggest that polyglutamylation levels could represent a predictor of therapeutic response to HD-MTX therapy with LV rescue in PCNSL. Furthermore, combination therapy with HD-MTX and agents inducing polyglutamylation might be useful for treating PCNSL.
